# Supplementary material for: Association of the abbreviated burn severity index with mortality in severely burned patients: A meta-analysis
Source: PLoS One. 2025 Feb 20;20(2):e0319199. doi: 10.1371/journal.pone.0319199 (PMC11841875; doi:10.1371/journal.pone.0319199)
Supplement: S2 Table — (DOCX) [file pone.0319199.s002.docx]

Supplementary table 2. Detailed results of NOS score.

| Author | Selection | | | | Comparability | Outcome measurement | | | Score |
| --- | --- | --- | --- | --- | --- | --- | --- | --- | --- |
|  | Representativeness of the exposed cohort | Selection of the non-exposed cohort | Ascertainment of exposure | Outcome of interest | Comparability of cohorts | Assessment of outcome | Time of follow-up | Adequacy of follow-up |  |
| Heng [22] | 1 | 1 | 1 | 1 | 0 | 1 | 1 | 0 | 6 |
| Pantet [23] | 1 | 1 | 1 | 1 | 1 | 0 | 1 | 1 | 7 |
| Yoon [24] | 1 | 1 | 1 | 1 | 0 | 1 | 1 | 0 | 6 |
| Barcellos [25] | 1 | 1 | 1 | 1 | 0 | 1 | 1 | 0 | 6 |
| Shahi [26] | 1 | 1 | 1 | 1 | 0 | 1 | 1 | 0 | 6 |
| Ding [27] | 1 | 1 | 1 | 1 | 0 | 0 | 1 | 1 | 6 |
| Chen [28] | 1 | 1 | 1 | 1 | 0 | 1 | 1 | 0 | 6 |
| Depret [29] | 1 | 1 | 1 | 1 | 0 | 1 | 1 | 0 | 6 |
| Zeng [30] | 1 | 1 | 1 | 1 | 0 | 1 | 1 | 0 | 6 |
| Lin [31] | 1 | 1 | 1 | 1 | 0 | 0 | 1 | 1 | 6 |
| Lin [32] | 1 | 1 | 1 | 1 | 0 | 1 | 1 | 1 | 7 |
| Tsolakidis [33] | 1 | 1 | 1 | 1 | 0 | 1 | 1 | 0 | 6 |
| Jiang [34] | 1 | 1 | 1 | 1 | 0 | 1 | 1 | 0 | 6 |
| Niculae [35] | 1 | 1 | 1 | 1 | 0 | 0 | 1 | 1 | 6 |
| Nitescu [36] | 1 | 1 | 1 | 1 | 0 | 1 | 1 | 0 | 6 |
| Christ [37] | 1 | 1 | 1 | 1 | 0 | 0 | 1 | 1 | 6 |

NOS: Newcastle-Ottawa Scale.
