# Supplementary material for: Association of the abbreviated burn severity index with mortality in severely burned patients: A meta-analysis
Source: PLoS One. 2025 Feb 20;20(2):e0319199. doi: 10.1371/journal.pone.0319199 (PMC11841875; doi:10.1371/journal.pone.0319199)
Supplement: Attachment — Submitted filename: response to comments.docx [file pone.0319199.s004.docx]

**Response to Editor: (Second round)**

1. Thank you for responding to our latest query. Before we can proceed with your manuscript, please could you provide the following details:
1) In the PRISMA flowchart, you specify that originally 611 records were screened for this study. Please provide a numbered table of all those 611 studies identified in the literature search, including those that were excluded from the analyses. For every excluded study, the table should list the reason(s) for exclusion. If any of the included studies are unpublished, include a link (URL) to the primary source. An example for how we would expect a table like this to look like can be found here: https://journals.plos.org/plosone/article?id=10.1371/journal.pone.0313866#sec017 (S2 file)
**Answer:** We have updated the Supplementary table 1 to list all 741 publications and reasons for exclusion.

2) Please also update the methods section of your manuscript with an explanation of how missing data were handled.

**Answer:** In the exclusion, we added the sentence: “4) studies without the OR and 95% CI for pooled analysis.”. And also “Furthermore, for missing data except for the OR and 95% CI, we presented them as “not reported (NR)”.”

3) Please provide a table showing the completed risk of bias and quality/certainty assessments for each study or outcome.

**Answer:** We have added the supplementary table 2 to show the completed risk of bias and quality/certainty assessments for each study or outcome.

**Response to Academic Editor:**

**Question 1:** In short, you will see a number of suggestions that are really needed to improve this story for acceptance. Particular attention to the comments of Reviewer 1 and Reviewer 2 are needed. Overall, more in depth analysis of the ASBI needs to be done, and what stands this work apart from previous studies on the ASBI is needed.

**Answer:** Dear editor, many thanks for your valuable comments and nice work. We sincerely appreciate the opportunity to revise our manuscript. We have carefully modified our manuscript according to the comment by all reviewers. Besides, we also discussed this issue in the manuscript to highlight the necessity of this study as follows: “The ABSI score, introduced in 1982, has been widely utilized as a prognostic tool for assessing mortality risk in burn patients [14]. However, over the past few decades, the validity of the ABSI score has been questioned because of advancements in burn care and the emergence of more sophisticated prognostic tools. One of the main criticisms is that the original score was developed based on patient data from a different era, with medical practices and outcomes that may not fully align with contemporary standards. For example, improvements in intensive care, fluid resuscitation, infection control, and surgical interventions have significantly enhanced patient survival, particularly among those with severe burns [46]. Additionally, some studies have suggested that the weight of certain variables in the ABSI score, such as sex or age, may not reflect the same level of prognostic significance in modern clinical settings [47]. Despite these criticisms, recent studies have reaffirmed the utility of the ABSI score, particularly in resource-limited settings or as a baseline comparison for newer predictive models [48]. These findings underscore the importance of continuously validating and updating prognostic tools such as the ABSI to ensure their relevance in guiding clinical decision-making and improving patient outcomes.”

**Question 2:** Additionally, your conclusions need to be updated. Presumably, this score includes age, TBSA, and inhalation injury, as was developed over 4 decades ago. Your conclusion that "ABSI could serve as a novel and reliable prognostic indicator in severely burned patients" is not true because the ASBI is not novel. Was there any comparison to other scoring systems (e.g., Baux)? Moreover, your conclusion that "patients with an elevated ABSI are exposed to increased risk of death." is also not new- age and TBSA have been known predictors of mortality for nearly 70 years. As such, I encourage your new conclusions to better highlight the uniqueness of this work, and what it brings to the field.

**Answer:** Dear editor, thanks for your valuable question. We have carefully revised our conclusion as request. Conclusion in the abstract: “The ABSI serves as a reliable prognostic indicator in severely burned patients, and patients with an elevated ABSI are at increased risk of death.”. Conclusion at the end of the manuscript: “The ABSI serves as a reliable prognostic indicator in patients with severe burns. Unlike previous studies, this meta-analysis provides robust evidence for the clinical utility of the ABSI. Our findings reinforce that patients with elevated ABSI scores are at an increased risk of mortality, which emphasizes the importance of this indicator in risk stratification and in guiding treatment prioritization in modern burn care. However, due to the limitations of our meta-analysis and in the included studies, more high-quality studies are still needed to verify our findings.” Furthermore, about the comparison between ABSI and other scoring systems, we have addressed this issue in the limitation part: “Fourth, we did not compare the prognostic value of the ABSI with that of other similar indicators, such as the Baux score, which is also difficult to achieve in meta-analyses because of the lack of original data.”

**Response to Journal editor:**

When submitting your revision, we need you to address these additional requirements.

1. Please ensure that your manuscript meets PLOS ONE's style requirements, including those for file naming. The PLOS ONE style templates can be found at

https://journals.plos.org/plosone/s/file?id=wjVg/PLOSOne_formatting_sample_main_body.pdf and

<https://journals.plos.org/plosone/s/file?id=ba62/PLOSOne_formatting_sample_title_authors_affiliations.pdf>.

**Answer:** We have revised the manuscript according to the PLOS ONE’s style requirements.

2. Thank you for stating the following in the Acknowledgments Section of your manuscript:

“This work was funded by the Key Projects of Science and Technology Department of Sichuan Province(2024YFFK0063).”

We note that you have provided additional information within the Acknowledgements Section that is not currently declared in your Funding Statement. Please note that funding information should not appear in the Acknowledgments section or other areas of your manuscript. We will only publish funding information present in the Funding Statement section of the online submission form.

Please remove any funding-related text from the manuscript and let us know how you would like to update your Funding Statement. Currently, your Funding Statement reads as follows:

“This work was funded by the Key Projects of Science and Technology Department of Sichuan Province(2024YFFK0063).”

Please include your amended statements within your cover letter; we will change the online submission form on your behalf.

**Answer:** We have deleted the funding statement from the manuscript and provided corresponding information in the online submission. Cover letter was also updated.

3. PLOS requires an ORCID iD for the corresponding author in Editorial Manager on papers submitted after December 6th, 2016. Please ensure that you have an ORCID iD and that it is validated in Editorial Manager. To do this, go to ‘Update my Information’ (in the upper left-hand corner of the main menu), and click on the Fetch/Validate link next to the ORCID field. This will take you to the ORCID site and allow you to create a new iD or authenticate a pre-existing iD in Editorial Manager.

**Answer:** The ORCID for the corresponding author has been updated.

4. Your ethics statement should only appear in the Methods section of your manuscript. If your ethics statement is written in any section besides the Methods, please move it to the Methods section and delete it from any other section. Please ensure that your ethics statement is included in your manuscript, as the ethics statement entered into the online submission form will not be published alongside your manuscript.

**Answer:** The ethics statement has been moved to the Methods section.

5. We note that your Data Availability Statement is currently as follows: [All data used in this work have been presented in the manuscript.]

Please confirm at this time whether or not your submission contains all raw data required to replicate the results of your study. Authors must share the “minimal data set” for their submission. PLOS defines the minimal data set to consist of the data required to replicate all study findings reported in the article, as well as related metadata and methods (https://journals.plos.org/plosone/s/data-availability#loc-minimal-data-set-definition).

For example, authors should submit the following data:

- The values behind the means, standard deviations and other measures reported;

- The values used to build graphs;

- The points extracted from images for analysis.

Authors do not need to submit their entire data set if only a portion of the data was used in the reported study.

If your submission does not contain these data, please either upload them as Supporting Information files or deposit them to a stable, public repository and provide us with the relevant URLs, DOIs, or accession numbers. For a list of recommended repositories, please see https://journals.plos.org/plosone/s/recommended-repositories.

If there are ethical or legal restrictions on sharing a de-identified data set, please explain them in detail (e.g., data contain potentially sensitive information, data are owned by a third-party organization, etc.) and who has imposed them (e.g., an ethics committee). Please also provide contact information for a data access committee, ethics committee, or other institutional body to which data requests may be sent. If data are owned by a third party, please indicate how others may request data access.

**Answer:** The Data Availability Statement has been updated as follows: “All data used in this work have been presented in the Supplementary Data File.” Meanwhile, the data used in this meta-analysis has been uploaded.

6. As required by our policy on Data Availability, please ensure your manuscript or supplementary information includes the following:

A numbered table of all studies identified in the literature search, including those that were excluded from the analyses.

For every excluded study, the table should list the reason(s) for exclusion.

If any of the included studies are unpublished, include a link (URL) to the primary source or detailed information about how the content can be accessed.

A table of all data extracted from the primary research sources for the systematic review and/or meta-analysis. The table must include the following information for each study:

Name of data extractors and date of data extraction

Confirmation that the study was eligible to be included in the review.

All data extracted from each study for the reported systematic review and/or meta-analysis that would be needed to replicate your analyses.

If data or supporting information were obtained from another source (e.g. correspondence with the author of the original research article), please provide the source of data and dates on which the data/information were obtained by your research group.

If applicable for your analysis, a table showing the completed risk of bias and quality/certainty assessments for each study or outcome.  Please ensure this is provided for each domain or parameter assessed. For example, if you used the Cochrane risk-of-bias tool for randomized trials, provide answers to each of the signalling questions for each study. If you used GRADE to assess certainty of evidence, provide judgements about each of the quality of evidence factor. This should be provided for each outcome.

An explanation of how missing data were handled.

This information can be included in the main text, supplementary information, or relevant data repository. Please note that providing these underlying data is a requirement for publication in this journal, and if these data are not provided your manuscript might be rejected.

**Answer:** We have revised the manuscript as request. Notably, we uploaded a supplementary table listing the reasons for exclusion after reviewing the full texts.

**Response to Reviewer #1:**

This manuscript analyzes the prognostic relevance of the ABSI score based on 16 studies published between 2015 and 2024. The statistical analysis is thorough and the results are presented in a clear and understandable form. However, the overall quality of the text lacks in multiple areas.

**Question 1:** Overall, many of the chosen formulations are inadequate and articles are often missing, which makes the text not very pleasant to read.

**Answer:** Dear reviewer, thanks for your valuable comments. We have carefully checked and revised the manuscript thoroughly. Besides, this manuscript has been edited by the AJE service with the verification code 51D0-8C70-A160-CA8E-72A8.

**Question 2:** The authors claim, that the ABSI score could possibly be used as a novel prognostic score, which doesn't make any sense as the score was introduced in 1982 and has been in use ever since. It is true, that the relevance of the score has been questioned in the past, however, there are multiple (very recent) studies which test the score for its validity. However, the authors repeatedly claim in this manuscript that the ABSI score is novel, which could be very misleading for readers.

**Answer:** Dear reviewer, sorry for the worry description. We have carefully revised the manuscript. In the introduction section, we indicated that the ABSI was developed in 1982, cited the reference and also reintroduced the necessity of conducting this study as follows: “The abbreviated burn severity index (ABSI), which was introduced in 1982, is a prognostic indicator that is based on sex, age, inhalation injury, occurrence of third-degree burns and total burned area [14]. In recent decades, this tool has been investigated in several studies since 2015 and is believed to have some prognostic value in severely burned patients. However, the results of these studies varied, and the clinical value of the ABSI in predicting mortality in patients with severe burns still lacks high-level evidence. Therefore, for the first time, this meta-analysis aimed to further clarify the association between the ABSI and the risk of mortality in patients with severe burns”

**Question 3:** Furthermore, the authors don't provide enough references for the claims in the introduction as well as the discussion. Following claims need to be undermined with references:
Introduction:
- Young children and the elderly, due to thinner skin and weaker immune function, tend to have poorer outcomes when faced with burns of the same severity. (??)
- Additionally, underlying conditions such as diabetes, heart disease, and renal failure can impair the body's ability to recover from burns, leading to a poorer prognosis. (e.g. Resch A, Neumueller A, Christ A, Staud C, Hacker S. Chronic kidney disease and cardiovascular disease reduce survival rates after burn injury: A retrospective study over 20 years.)
- Severe burn patients are prone to infections, especially with extensive burns, and infection is one of the leading causes of death in these cases.
- However, due to weaker immunity and lower treatment compliance, children may have a worse prognosis compared to adult patients in the same situation. (The ABSI score is especially relevant for severely burned patients, who require intensive care treatment. There is no such thing as "lower treatment compliance" in children in ICU's.)
**Answer:** Dear reviewer, many thanks for your valuable comments. We have carefully added corresponding references to support these claims. Besides, this sentence “However, due to weaker immunity and lower treatment compliance, children may have a worse prognosis compared to adult patients in the same situation. (The ABSI score is especially relevant for severely burned patients, who require intensive care treatment” has been deleted.

**Question 4**: In the materials and methods section the authors write "Studies met following criteria were included: 1) severely burned patients with the degree II burns ≥30% or degree II burns ≥10% of the total body surface area,...." which doesn't make any sense and needs to be rewritten and clarified.
**Answer:** Thanks for your valuable question. We have revised this sentence as follows: “patients included had severe burns, which are defined as those requiring transfer to a designated burn center for treatment according to established guidelines [16, 17]”.

**Question 5:** Overall the discussion is weak and lacks in relevant information about the ABSI score and why it's validity has been questioned over the past decades.

**Answer:** Dear reviewer, thanks for your valuable question. We have carefully added the discussion about this issue as follows: “The ABSI score, introduced in 1982, has been widely utilized as a prognostic tool for assessing mortality risk in burn patients [14]. However, over the past few decades, the validity of the ABSI score has been questioned because of advancements in burn care and the emergence of more sophisticated prognostic tools. One of the main criticisms is that the original score was developed based on patient data from a different era, with medical practices and outcomes that may not fully align with contemporary standards. For example, improvements in intensive care, fluid resuscitation, infection control, and surgical interventions have significantly enhanced patient survival, particularly among those with severe burns [46]. Additionally, some studies have suggested that the weight of certain variables in the ABSI score, such as sex or age, may not reflect the same level of prognostic significance in modern clinical settings [47]. Despite these criticisms, recent studies have reaffirmed the utility of the ABSI score, particularly in resource-limited settings or as a baseline comparison for newer predictive models [48]. These findings underscore the importance of continuously validating and updating prognostic tools such as the ABSI to ensure their relevance in guiding clinical decision-making and improving patient outcomes.”

**Response to Reviewer #2:**

**Question 1:** The conclusion should be a statement in the affirmative rather than subjuctive, i.e. 'ABSI serves as a reliable prognostic indicator for mortality in burned patients'. By the way, it is not novel,... This phrase should be deleted.

**Answer:** Dear reviewer, thanks for your valuable question. We have carefully revised the conclusion as request: Conclusion in the abstract: “The ABSI serves as a reliable prognostic indicator in severely burned patients, and patients with an elevated ABSI are at increased risk of death.”. Conclusion at the end of the manuscript: “The ABSI serves as a reliable prognostic indicator in patients with severe burns. Unlike previous studies, this meta-analysis provides robust evidence for the clinical utility of the ABSI. Our findings reinforce that patients with elevated ABSI scores are at an increased risk of mortality, which emphasizes the importance of this indicator in risk stratification and in guiding treatment prioritization in modern burn care. However, due to the limitations of our meta-analysis and in the included studies, more high-quality studies are still needed to verify our findings.”

**Question 2:** Age is included as a major factor in all useful predictors, which are burn size and age and should be introduced together.

**Answer:** Thanks for your comment. We have carefully modified this section to introduce them together as follows: “The most commonly used indicators are the total body surface area (TBSA) burned, burn depth and age [6]. Generally, larger burn areas (greater than 30%) and deeper burns, such as deep partial-thickness and full-thickness burns (third-degree burns), are associated with worse outcomes [6]. In addition, young children and elderly individuals, due to their thinner skin and weaker immune function, tend to have poorer outcomes when experiencing burns of the same severity [7, 8]. If a patient has inhalation injuries, such as those caused by the inhalation of hot smoke or chemical gases, and airway damage ensues, the prognosis worsens, which results in a higher mortality rate [9]”

**Question 3:** ABSI has been around for a long time, and the major inclusion over age and burn size alone (a.k.a. Baux score) is inhalation injury, which goes back and forth. The paper should really include an analysis of Baux score too. Is ABSI better than the Baux score in a meta-analysis?

**Answer:** Thanks for your valuable question. We have added the introduction about ABSI in the introduction section. Actually, in this type of study, it is hard to compare the prognostic value of ABSI with other indicators due to the lack of original data. We have added this issue in the limitation part as follows: Fourth, we did not compare the prognostic value of the ABSI with that of other similar indicators, such as the Baux score, which is also difficult to achieve in meta-analyses because of the lack of original data.”

**Question 4:** How exactly were the studies selected? Was inclusion confirmed by more than one independent assessor? Were PRISMA guidelines used?

**Answer:** Thanks for your comments. Actually, the literature search, selection, data extraction, and methodological quality assessment were all performed by two authors independently, which has been added in the manuscript “Two investigators independently performed the literature search, selection, data extraction, and methodological quality assessment, and all disagreements were resolved by team discussion.”. Besides, as we mentioned in the methods part, “The current meta-analysis was performed according to the Preferred Reporting Items for Systematic Reviews and Meta-Analyses 2020 [15].”

**Question 5:** What exactly does 'elevated ABSI' mean? The statistical methodology should be much clearer. This should probably be done with categorical variables. For instance, an OR of 68 is massive and not all that instructive. Further, most evidence would suggest that this is non-linear which is not really reflected in the analysis.

**Answer:** Dear reviewer, thanks for your professional question. Actually, in most studies, the ABSI was treated as a continuous variable as presented in table 1. Therefore, it is hard to accurately defined the “elevated ABSI”, which is a common limitation of meta-analyses. Besides, we have added the discussion about this issue in the manuscript as follows: “In most of the included studies, the ABSI was treated as a continuous variable, whereas in some studies, it was categorized as a binary variable. Although we performed a subgroup analysis to address this issue, for better clinical application of the ABSI in patient risk assessment, more precise risk stratification may still be needed.”

**Question 6:** What is the range of ABSI? The amount of data available suggest a much more detailed analysis.

**Answer:** Thanks for your question. As mentioned in the inclusion criteria, we introduced the scoring criteria of ABSI and also added the range of ABSI: “and it ranged from 2 to 19”. Actually, we tried to perform more subgroup analyses based on the age, grouping method and source of OR. However, we were unable to conduct more detailed subgroup analysis due to the lack of original data as mentioned in the limitation section “Third, we were unable to conduct more subgroup analyses based on other important parameters, such as the type of burn and cutoff value of the ABSI, due to the lack of original data.”

**Response to Reviewer #3**:

Dear author Congratulations about this work
I suggest that you correct line 15 degree II to Degree III > 10%
And in discussion if you found another characteristics related to mortality beyond ABSI ( as face, inhalation) i suggest that you commented
**Answer:** Thanks for your valuable comments. For the first question, this sentence has been carefully corrected to as follows according to the comments by reviewer 1: “patients included had severe burns, which are defined as those requiring transfer to a designated burn center for treatment according to established guidelines [16, 17]”. For the second question, we have added the discussion about this issue in the limitation section as follows: “. Fourth, we did not compare the prognostic value of the ABSI with that of other similar indicators, such as the Baux score, which is also difficult to achieve in meta-analyses because of the lack of original data.”

**Response to Reviewer #4:**

The article entitled "Association of abbreviated burn severity index with mortality in severely burned patients: a meta-analysis" aimed to validate a commonly used tool in burn research to predict mortality, performed as a meta-analysis of data from around the world. The aims of this study are important to both research and clinical practice. From a technical standpoint the meta-analysis is performed well.
Suggestions:
**Question 1:** Consider discussing the ABSI more – even showing the formula – so that the general readership understands how it works and how it is applied

**Answer:** Dear reviewer, thanks for your valuable question. Actually, in the inclusion criteria, we have introduced the scoring criteria of ABSI in detail. “ABSI was evaluated according to the sex (female: score 1; male: score 0), age (0-20-year-old: score 1; 21-40-year-old: score 2; 41-60-year-old: score 3; 61-80-year-old: score 4; 81-100-year-old: score 5), inhalation injury (with: score 1; without: score 0), degree III burn (with: score 1; without: score 0) and total burned area (1%-10%: score 1; 11%-20%: score 2; 21%-30%: score 3; 31%-40%: score 4; 40%-50%: score 5; 50%-60%: score 6; 60%-70%: score 7; 70%-80%: score 8; 80%-90%: score 9; 90%-100%: score 10) and it ranged from 2 to 19”

**Question 2:** At minimum, the original paper that introduced the ABSI (Tobiasen J, Hiebert JM, Edlich RF. The abbreviated burn severity index. Ann Emerg Med. 1982;11:260–2.) should be referenced.

**Answer:** Thanks for your valuable question. We have carefully introduced the ABSI in the introduction and cited this reference. “Abbreviated burn severity index (ABSI) is a prognostic indicator based on the sex, age, inhalation injury, degree III burn and total burned area, which was introduced in 1982 [14]”

**Question 3:** You report odds ratios for the ABSI. As a clinician who would use this tool in practice, this is not a particularly useful statistic. I would consider elements such as sensitivity, specificity, PPV, NPV, and probably most helpful, ROC curves, to help communicate the accuracy of the ABSI. There are at least 10 tools to predict mortality in burns, all with pros and cons. The paper should ideally make this as clinically relevant as possible.

**Answer:** Dear reviewer, thanks for your question and we completely agree with your opinion. However, it is unable to apply these methods in meta-analyses because of the lack of original data. We also discussed this issue in the limitation section.
